# Common pitfalls, and how to avoid them, in child and adolescent psychopharmacology: Part I

**DOI:** 10.1177/02698811241239582

**Published:** 2024-03-18

**Authors:** Samuele Cortese, Frank MC Besag, Bruce Clark, Chris Hollis, Joe Kilgariff, Carmen Moreno, Dasha Nicholls, Paul Wilkinson, Marc Woodbury-Smith, Aditya Sharma

**Affiliations:** 1 Centre for Innovation in Mental Health, School of Psychology, Faculty of Environmental and Life Sciences, University of Southampton, Southampton, UK; 2 Solent NHS Trust, Southampton, UK; 3 Hassenfeld Children’s Hospital at NYU Langone, New York University Child Study Center, New York City, NY, USA; 4 DiMePRe-J-Department of Precision and Regenerative Medicine-Jonic Area, University of Bari ‘Aldo Moro’, Bari, Italy; 5 UCL School of Pharmacy, London, UK; 6 East London Foundation NHS Trust, Bedfordshire, UK; 7 National Specialist Clinic for Young People with OCD, BDD and Related Disorders, South London and Maudsley NHS Foundation Trust, London, UK; 8 Department of Child and Adolescent Psychiatry, Nottinghamshire Healthcare NHS Foundation Trust, Queen’s Medical Centre, Nottingham, UK; 9 National Institute of Mental Health (NIHR) MindTech Medtech Co-operative, Institute of Mental Health, Nottingham, UK; 10 NIHR Nottingham Biomedical Research Centre, Mental Health & Technology Theme, Institute of Mental Health, Nottingham, UK; 11 Department of Child and Adolescent Psychiatry, Institute of Psychiatry and Mental Health, Hospital General Universitario Gregorio Marañón, IiSGM, CIBERSAM, ISCIII, School of Medicine, Universidad Complutense, Madrid, Spain; 12 Division of Psychiatry, Imperial College London, London, UK; 13 NIHR ARC Northwest London, London, UK; 14 School of Clinical Medicine, University of Cambridge, Cambridge, Cambridgeshire, UK; 15 Cambridgeshire and Peterborough NHS Foundation Trust, Cambridge, UK; 16 Biosciences Institute, Newcastle University, Newcastle upon Tyne, UK; 17 Academic Psychiatry, Translational and Clinical Research Institute, Newcastle University, Newcastle upon Tyne, UK; 18 Specialist Adolescent Mood Disorders Service (SAMS), Cumbria, Northumberland, Tyne and Wear NHS Foundation Trust, Newcastle upon Tyne, UK

**Keywords:** Psychopharmacology, children, adolescents, young people, pitfalls, expert opinion

## Abstract

As Faculty of the British Association for Psychopharmacology course on child and adolescent psychopharmacology, we present here what we deem are the most common pitfalls, and how to avoid them, in child and adolescent psychopharmacology. In this paper, we specifically addressed common pitfalls in the pharmacological treatment of attention-deficit/hyperactivity disorder, anxiety, bipolar disorder, depression, obsessive-compulsive disorder and related disorders, and tic disorder. Pitfalls in the treatment of other disorders are addressed in a separate paper (part II).

## Introduction

As Faculty of the British Association for Psychopharmacology (BAP) course on child and adolescent psychopharmacology, we previously published a paper (Cortese et al., 2023) reporting the most common questions we have been asked in recent editions of the course, alongside evidence-based and/or expert-informed answers. Here, based on our experience during the course, we have selected what we deem are the most common pitfalls, and how to avoid them, in child and adolescent psychopharmacology, focusing on attention-deficit/hyperactivity disorder (ADHD), anxiety, bipolar disorder, depression, obsessive-compulsive disorder and related disorders, and tic disorder. We have grouped the pitfalls by disorder to which they refer, in alphabetical order. Pitfalls in relation to the treatment of other disorders (autism and intellectual disability, eating disorders, neuropsychiatric correlates of epilepsy and psychosis) are addressed in a separate paper (part II).

## Attention-deficit/hyperactivity disorder

### Switching to equivalent doses of long-acting formulations of methylphenidate

When switching patients from immediate- or extended-release methylphenidate (which acts by inhibiting the reuptake of dopamine and norepinephrine) to a long-acting methylphenidate formulation, prescribers should avoid a simple equivalence based on the total dose. Rather, they should use the immediate-release component of each formulation as the reference. For instance, when switching from 20 mg of Medikinet XL^®^ (methylphenidate extended release, ER) (50% immediate release: 10 mg) to OROS Concerta XL^®^, 45 mg of OROS Concerta XL^®^ (22% immediate release: 9.9 mg) would give the equivalent immediate-release dose (Coghill et al., 2013). Along the same line of reasoning, 40 mg of Equasym XL^®^ (methylphenidate extended release, ER) (30% immediate release: 12 mg) would be equivalent to OROS Concerta XL^®^ 54 mg and Equasym XL^®^ 50 mg to, roughly, OROS Concerta XL^®^ 72 mg.

### Optimising the treatment

While the maximum licensed dose of methylphenidate for children (except for osmotic release and prolonged release formulations, see below) is 60 mg/day in many countries, some guidelines (e.g. those from the Canadian ADHD Resource Alliance (CADDRA), caddra.ca) and other documents recommend higher doses (Cortese, 2020). For instance, the British National Formulary (BNF; Joint Formulary Committee, 2022) recommends a dose of up to 90 mg/day, under the direction of a specialist. For osmotic-release and prolonged-release formulations of methylphenidate, the maximum license dose is 54 mg/day, but the BNF mentions a maximum of 108 mg/day for Concerta XL^®^ in children (as well as in adults).

Some prescribers may fail to optimise the dose of treatment to reach the maximum benefit with the highest tolerated dose, being satisfied with a moderate improvement in the severity of the symptoms. Meta-analytic evidence based on flexible-dose trials for both methylphenidate and amphetamines shows increased efficacy and reduced likelihood of discontinuations for any reason with increasing stimulant doses, up to the maximum FDA-licensed dose (Farhat et al., 2022). Also, meta-analytic evidence in adults (not available in children) shows that, at the group level, doses beyond the licensed ones are generally not associated with a favourable benefit–risk profile, even though it is important to appreciate that individual patients may benefit from, and tolerate well, doses beyond the licensed ones (Farhat et al., 2024). While it should not be a standard practice, using doses beyond the maximum recommended ones could be considered when the patient has presented with a partial response, there is only some degree of improvement at the maximum recommended dose, and tolerability is good. It should be noted that, in some countries, special authorisation may be necessary for doses exceeding the maximum licensed amount, subject to local regulations.

### Drug holidays

An important issue is around adherence to medication (Baweja et al., 2021). Due to concerns about the side effects of stimulants, some prescribers may consider advising stopping the treatment during the weekend. However, to our knowledge, to date, only one randomised trial showed a trend (*p* = 0.08) for an association between drug holidays on the weekend and less interference with appetite (Martins et al., 2004). The European ADHD Guidelines Group (Cortese et al., 2013) advised that the risk–benefit balance of drug holidays during weekends must be taken into account and better investigated. Evidence on the beneficial effects on appetite of stopping medication during longer drug holidays (e.g. summer holidays) to allow for catch-up growth is also mixed (Faraone et al., 2008). Therefore, discontinuing stimulants during the weekend should be considered in cases where there is a clear benefit from the stimulant but serious concerns around appetite reduction, even though simulant discontinuation could not be enough to allow catching up the weight. If, despite the implementation of the previous management strategies, weight and/or height values are below critical thresholds, a referral to the paediatric endocrinologist or growth specialist is recommended (Cortese et al., 2013).

Note: General aspects of the psychopharmacology of ADHD are covered elsewhere (e.g. Cortese et al., 2018; Cortese 2020; Faraone et al., 2021).

## Anxiety and depression

### Is the diagnosis correct?

A correct and complete diagnostic assessment is essential. We have evidence for what works for depressive and anxiety disorders (cognitive behavioural therapy (CBT) for both, selective serotonin reuptake inhibitors (SSRIs) for both (but probably just robust evidence for fluoxetine in depression) and interpersonal psychotherapy (IPT) for depression only (James et al., 2020; Strawn et al., 2015; Zhou et al., 2020)), but no evidence that those same treatments work for other disorders/problems, for example, situational sadness. So, prescribers should ensure that diagnostic criteria are met.

Particular caution needs to be taken around personality disorders (in particular emotionally unstable personality disorder (EUPD), also referred to as borderline personality disorder). We note that the diagnosis of personality disorders in adolescence is controversial, with some practitioners/researchers endorsing this diagnosis, and others using the term *emerging personality disorder* (Elvins and Kaess, 2022). Young people with EUPD or *emerging personality disorder* may appear to have depression during the first assessment, as they are most likely to present when their mood is low. A careful assessment is essential. If young people diagnosed with a depressive disorder do not respond to first-line treatment, the prescriber should go back and review the diagnosis and consider personality disorder/*emerging personality disorder* (which tends to become more obvious the longer a young person is treated).

### Are we addressing all the problems?

A complete diagnostic formulation is essential. Basic treatments aimed solely at depression or anxiety are unlikely to be effective if there are significant co-morbidities. For instance, if ADHD is causing educational failure, leading to low self-esteem, then this needs to be addressed alongside treatment for depression. Another example is when CBT for depression ignores the primary anxiety disorder, which is unlikely to lead to the resolution of the depression.

We must also address social factors that contribute to depression – for example, if a young person is being bullied and becomes depressed, an SSRI will not stop the bullying which makes them feel worthless. We must enquire about social factors and do our best to address them and revisit what social stresses there may be if a young person does not respond to treatment. One should bear in mind that young people may not disclose challenging events in their lives (particularly abuse) during the first assessment, but they may feel more comfortable with the practitioner in subsequent sessions.

### Are they really ‘treatment resistant’?

Pharmacologically treatment-resistant depression is defined, in adults, by at least two prior treatment failures with adequate dose and duration (Gaynes et al., 2020). Similarly, pharmacologically treatment-resistant anxiety has been recently defined in adults as at least two separate failed full trials of pharmacological monotherapy with first-line agents approved for those disorders by the US Food and Drug Administration (FDA) or the European Medicines Agency or other equivalent regulatory agencies, and recommended by guidelines (Domschke et al., 2024). Treatment-resistant depression and anxiety in children and young people are difficult to treat, with little research evidence to guide the practitioner. However, we need to review the history of treatment before concluding there is treatment resistance. Notably, significant numbers of people stop antidepressants when they have minor side effects which may have worn off if they had persevered. The amount of time needed to wait until side effects go away is very variable, but we would advise patients to persevere for a month if side effects are tolerable, before stopping.

It may be worth going back to re-try the antidepressant with psychoeducation and encouragement to persevere. A slower dose titration may also reduce the impact of side effects. It is also appropriate to increase the dose to BNF dose limits if tolerated. It is important to discuss with the patient and their families the risks and benefits of this strategy, and how this is outside the license for adolescents, but is an acceptable treatment for adults, and often used in adolescents.

A particularly concerning side effect of SSRIs is increased suicidal thoughts. These have only been demonstrated to be significantly higher than placebo in randomised controlled trials of depression, not of anxiety (Bridge et al., 2007), and indeed the UK 2003 MHRA and USA 2004 FDA Black Box warnings only applied to adolescent depression, not anxiety disorders (https://journalofethics.ama-assn.org/article/antidepressants-and-fdas-black-box-warning-determining-rational-public-policy-absence-sufficient/2012-06#:~:text=The%20MHRA%27s%202003%20recommendation%2C%20based,randomized%20clinical%20trials%20%5B1%5D). However, negative results in anxiety may reflect a lower baseline risk and fewer total participants in trials. Hence, prescribers need to watch carefully for suicidality. Due to the severe potential consequences of suicidal thoughts, many prescribers and families would choose not to continue SSRIs if they emerged.

We note that it can be even more difficult to judge treatment resistance for psychological therapy. To determine true treatment resistance, we need to ensure the patient has good quality therapy at an adequate dose. This requires a fully accredited therapist, appropriate expert supervision of the therapist and an adequate number of sessions for the patient to try out techniques, as opposed to having one session and deciding it was ‘rubbish’. In addition, patients may find it hard to get on well with therapist A but get on well with (and be prepared to try therapy with) therapist B.

General guidance on the pharmacological treatment of anxiety (provided, for instance, in Patel et al., 2018) and depressive disorders (e.g. Vitiello and Ordóñez, 2016) in children and adolescents, mentioning key studies in the field such as the Treatment for Adolescents with Depression Study (March et al., 2004) or the Treatment of SSRIs-resistant depression in adolescence (Brent et al., 2008), is beyond the scope of the current paper.

## Bipolar disorder

### Prescribing subtherapeutic doses of mood-stabilising agents

Prescribers often report ‘breakthrough’ episodes, with symptoms emerging during treatment, despite good adherence to the regime advised. In such cases, frequently, the dose of psychotropic medication being prescribed is subtherapeutic, such as, for instance, when quetiapine (receptor antagonist (D2 and 5-HT2)) 25 mg in the morning and 50 mg at night is prescribed. It is more likely in such cases that the index episode for which the medication was being prescribed self-remitted and the improvement was *not* associated with medication. Therefore, prescribers should familiarise themselves with therapeutic doses of psychotropic medications and use them appropriately whilst monitoring for both efficacy and side effects.

### Continuing antidepressants in the case of treatment-emergent affective switch

The use of antidepressants to treat unipolar depression, anxiety disorders and OCD in children and adolescents may be associated with treatment-emergent affective switch (TEAS) as described in our previous paper (Cortese et al., 2023). Prescribers often consider a gradual tapering of antidepressants to avoid a discontinuation syndrome. However, it would actually be best practice to stop antidepressants when TEAS is evident, as continued use of antidepressants even in small doses may perpetuate the TEAS.

### Management of bipolar depression

Bipolar depressive episodes are frequent in children and adolescents but are also frequently missed in clinical practice. When these require management with psychotropics, it is prudent to check adherence to medication and/or optimising the dose of medication. Antidepressants should be used cautiously to treat bipolar depressive episodes in children and specifically *always* with an appropriate mood stabilising agent(s) at a therapeutic dose (Goodwin et al., 2016). The use of antidepressants as monotherapy may be associated with a switch in polarity, emergence of mixed features and/or rapid cycling.

Note: General aspects of the treatment of bipolar disorder in children and young people are reported elsewhere (e.g. Singh et al., 2021).

## Obsessive-compulsive disorder and related conditions

### Insufficient evidence base

It is a common pitfall to think that the evidence base accurately covers the full range of obsessive and compulsive conditions. This is not the case. For instance, to date, there is no published evidence supporting the role of low-dose antipsychotic augmentation in body dysmorphic disorder (BDD) nor is any clear evidence that SSRI medications are helpful in the treatment of trichotillomania (Farhat et al., 2020). It is important therefore to be clear about the dominant aspect of the mental state in the child and make decisions on the evidence base, where it exists and, in turn, being clear where there is a lack of evidence or indeed evidence that medication is not likely to be of benefit.

### Co-morbidity in obsessive-compulsive disorders

The issue of co-morbidity in the presentation requires careful thought. A common pitfall in treatment planning is not comprehensively assessing the full clinical presentation and then missing opportunities to design the best treatment plan. Co-morbid mood (more than 60%) and anxiety (more than 75%) diagnoses are extremely common in young people with obsessive-compulsive disorders (OCD) (Pampaloni et al., 2022). These co-morbidities may require careful additional consideration, as they will undoubtedly influence the choice and sequencing of pharmacological interventions for OCD (Lochner et al., 2014). As an example, whilst trichotillomania is unlikely to respond to treatment with SSRI medication, the presence of a co-morbid affective disorder might increase the likelihood of a better medication response, in a young person with trichotillomania.

The broader neurodevelopmental profile can help guide the best choice of medications, as well as their sequencing. For instance, a common pitfall can be to attempt to treat OCD with psychological therapy in a patient with co-morbid ADHD, without adequately addressing the impact of ADHD. This can render treatment much less effective in our experience. We therefore find that ensuring ADHD symptoms are adequately treated pharmacologically can be a helpful precursor to better treatment outcomes of OCD. Some young people, however, can become more focussed on their compulsions when treated for ADHD.

### Dose and duration

NICE guidelines encourage the adoption of the maximum tolerated dosing schedule for SSRI medication in the treatment of OCD (NICE, 2005), as high-dose treatment (i.e. 200 mg/day for sertraline) is likely to be needed to effect good clinical outcomes in the majority of patients within OCD. In our experience, one of the most common pitfalls is to use too low a dose of SSRI, for too short a duration, and to not carefully support young people and their families to increase to a high dose of SSRI medication.

We believe that this pitfall can be mitigated with careful explanation, which includes sharing good quality psychoeducational materials about SSRI and other medications. This is extremely important in building a therapeutic alliance, to then attempt the necessary increase towards high-dose SSRI treatment regimens. Another common pitfall is for patients and their families to seek early discontinuation of medication when experiencing early side effects. Instead, the approach should be towards a maximum tolerated dosing approach. We think this pitfall of early discontinuations can be avoided by, again, carefully counselling young people and their families. As a result, if any treatment-emergent side effects do occur, then the practitioner will have proactively counselled that these can commonly settle in a short period of time. Most importantly, the approach incorporates each treatment trial of SSRI medication for a minimum of 10–12 weeks at the highest tolerated dose (AACAP, 1998, NICE, 2005). Our experience of being clear about this is that it then avoids abrupt treatment discontinuations, with incumbent withdrawal side effects. With careful planning and explanation, patients and their families realise one can drop back to the last ‘maximum tolerated dose’ and continue the treatment trial, rather than feeling this medication does not suit and needs to be discontinued. It is important to have clinical oversight as to whether in these cases a lower dose of an SSRI is at such a low level, that it is unlikely to be efficacious. Clinical experience is necessary to decide, in this circumstance, if it will be better to cross-titrate to another SSRI medication, in the hope of achieving higher dosing and therefore a greater chance of a positive treatment response.

### Augmentation with antipsychotics

A further pitfall in relation to medication regime for OCD relates to the use of antipsychotic augmentation of SSRI treatment. The evidence suggests that low-dose antipsychotic augmentation benefits around one-third of adult patients with OCD (Bloch, 2006). This has not been replicated in studies in children and young people but is considered and supported under NICE guidelines (NICE, 2005). A common pitfall is then increasing the dose of antipsychotic medication to a higher dose, when not seeing any improvements in OCD patients. This lack of additional treatment response in augmented OCD patients can be expected in around two-thirds of patients. This pitfall can therefore be avoided by a clear explanation that the treatment trial of SSRI augmentation will be around 2 months in duration and with a clear remit to only try the low doses of antipsychotics, such as risperidone (D2, 5-HT2, and NE alpha 2 receptor antagonist) 0.5 mg, or up to aripiprazole (D2 and 5-HT1A receptor partial agonist) 5 mg daily. As mentioned, we do not recommend augmenting SSRI treatment with antipsychotics in young people with BDD. This is despite the high incidence of delusional intensity appearance-related beliefs, in patients with BDD. Indeed, evidence is clear that delusional and non-delusional patients with BDD are just as likely to respond to SSRI treatments (Phillips and Hollander, 2008).

### Paediatric acute-onset neuropsychiatric syndrome

We note that, as standards of care are still being developed for Paediatric Acute-onset Neuropsychiatric Syndrome (PANS) and its treatment, and authoritative guidelines such as the NICE guidelines are not available, we have refrained from including here any recommendation on PANS. We look forward to additional high-level evidence to inform clinical decision-making. At the time of the writing, in the UK, the work of a PANS/PANDAS Working group is ongoing and there are no guidance and treatment recommendations to diagnose/treat PANS/PANDAD in the National Health System (NHS) (https://commonslibrary.parliament.uk/research-briefings/cdp-2023-0174/#:~:text=There%20is%20currently%20no%20guidance,and%20patients%20and%20their%20families).

## Tics

### Assessing the effectiveness of drug treatments

*Allow sufficient time*: The natural course of tics is a waxing (increasing) and waning (decreasing) pattern over weeks or months. Over hours and days, tics can also worsen due to, for example, anxiety, anger, excitement or tiredness. Tics commonly also wax during stressful periods such as starting the new school year or exams. A common pitfall is to prematurely assume treatment is effective when simply observing a natural waning phase, or conversely, that a drug treatment is ineffective when observing a natural waxing phase. To avoid these pitfalls, it is important to evaluate treatment effects over a sufficiently long period, usually 2–3 months, to compare peaks (waxing phases) and throughs (waning phases) before and after treatment initiation.

*Is the tic diagnosis correct?* Since the COVID-19 pandemic, there has been an unprecedented rise in functional tics, particularly in teenage girls (Pringsheim et al., 2023). Functional tics can be difficult to differentiate from a primary tic disorder and can also co-occur with primary tics. However, as functional tics are not responsive to standard tic medications – a common pitfall when failing to observe a tic treatment response at standard doses may be to continue dose escalation, thus causing increasing adverse effects (Malaty et al., 2022). Another common diagnostic pitfall is related to motor mannerisms and stereotypies which occur in autism and can mimic complex motor tics but are not responsive to tic treatments.

### Dosing

*Failing to ‘start low and go slow’*: When initiating medication, typically adverse effects occur before therapeutic benefits are seen – and can lead to premature discontinuation. This can be avoided by gradual titration; ‘starting low and going slow’. For example, initial syncope (due to postural hypotension) can be related to the initiation of clonidine (which stimulates postsynaptic alpha 2-adrenergic receptors) and guanfacine (which stimulates postsynaptic alpha 2A-adrenergic receptors) and sedation is potentially related to all medications for tics. These adverse effects are often encountered if the initial dose is too high. However, with careful low-dose initiation and slow titration, these adverse effects are much less likely to occur. Doses of 25 mcg of clonidine, 1 mg guanfacine and 0.5–1 mg aripiprazole once daily (Murphy et al., 2013) are suggested as appropriate initial doses, with titrations using a weekly, fortnightly or even monthly approach according to the response. However, the initial dose and the titration speed could differ according to body weight, age and other characteristics of the patient.

*Failing to achieve an adequate therapeutic dose*: When a medication has been initiated, it is important to titrate to recommended therapeutic doses. In the case of clonidine, this relates to an average daily dose of 3–5 mcg/kg (Taylor et al., 2021), for aripiprazole around 5–10 mg daily (Roessner et al., 2022) and for guanfacine between 1 and 4 mg in young children and up to 7 mg in older adolescents according to weight (Compendium, 2023). Often young people are sub-optimally medicated to reduce potential side effects. However, it is a common pitfall to conclude that a medication is ineffective, when in fact it has been used at a sub-therapeutic dose.

### Drug choice – is the drug correct?

Awareness of co-morbidities is important in determining the best drug choice. Commonly, young people with co-morbid tics and ADHD find noradrenergic agents (clonidine, guanfacine) beneficial for tic symptom control, whereas young people with anxiety disorders or OCD co-morbidities may find an approach using aripiprazole or treatment primarily targeting anxiety and/or OCD with an SSRI most beneficial in managing tic symptoms.

### Misidentifying new-onset tics as an adverse effect of stimulant medication for ADHD

Tic disorders occur in around 20% of young people with ADHD. However, the onset of tics (typically around 7–8 years of age) is later than the emergence of ADHD symptoms. Therefore, a common pitfall when observing new onset tics in children receiving stimulants for ADHD is to erroneously assume that the tics are a stimulant adverse effect (Hollis et al., 2016), leading to medication being stopped/swapped/reduced, when fact it is an emerging co-morbid tic disorder. If tics emerge very soon after starting stimulant medication – a dose reduction is warranted, to observe if tics reduce. However, if tics emerge after a stable period of ADHD treatment, and tics also require treatment, then augmentation with clonidine or guanfacine should be considered.

### Augmenting medication with behavioural therapy

It is a common pitfall to assume that medication should only be tried *after* using psychological approaches such as behavioural therapy. Whilst acknowledging in reality these resources are scarce, when available, it is not always beneficial to consider medication only after first attempting psychological therapies. Often barriers to successfully implementing these therapists can relate to the age or intellectual ability of the young person, co-morbidities such as ADHD and autism, family and environmental factors, and severity/impairment of the tics. For more severe tics, medication may reduce tic intensity/frequency and make behavioural techniques easier to implement. Notably, there is no current evidence to suggest that medication reduces the efficacy/effectiveness of behavioural interventions.
